# A giant ureteric calculus successfully removed by mini-endoscopic combined intrarenal surgery: A case report

**DOI:** 10.1016/j.eucr.2022.102270

**Published:** 2022-10-26

**Authors:** Pasin Limudomporn, Yada Phengsalae, Chinnakhet Ketsuwan

**Affiliations:** Division of Urology, Department of Surgery, Faculty of Medicine Ramathibodi Hospital, Mahidol University, Bangkok, Thailand

**Keywords:** Giant ureteric calculus, Mini-ECIRS

## Abstract

Giant ureteric calculi are extreme rare and associated with a subsequent decline in the function of the affected kidney. We report the case of a 58-year-old male with a huge opaque ureteral calculus found during a routine medical check-up. Computed tomography revealed an 11 × 12 × 67 mm^3^ ureteral stone at the right proximal ureter with mild hydronephrosis. The patient was treated successfully by mini-endoscopic combined intrarenal surgery, and the entire stone was retrieved. The patient recovered fully without additional complications.

## 1 Introduction

Giant ureteric calculi are extremely rare and typically diagnosed in stones greater than 5 cm in length or 50 g in weight. They may occur as a consequence of tuberculosis, ureteroceles, an ectopic ureter, a benign ureteral polyp, or in the absence of any anatomical abnormalities.^1^ Traditional approaches to treating giant ureteral stones are based on the function of the affected kidney and may necessitate either active stone removal or a simple nephroureterectomy.

As a consequence of breakthroughs in urologic interventional techniques and technologies, the ways to eradicate urolithiasis have increased. Miniaturized endoscopic combined intrarenal surgery (mini-ECIRS) is a novel therapeutic option that has become increasingly acceptable for the removal of a large or complex nephrolithiasis. To the best of our knowledge, this is the first clinical case reporting mini-ECIRS in a patient with a giant ureteric calculus.

## 2 Case report

**Fig. 1:**
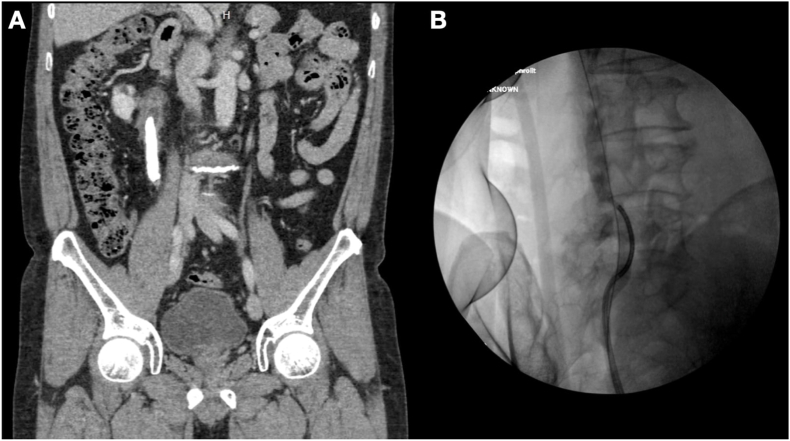
A, Computerized tomography scan showing the giant calculus; B, fluoroscopy showing the ureteric calculus that was relocated upward using a flexible ureteroscope.

A 58-year-old male was referred to our department with a large ureteral stone that was detected on routine medical examination. He was in excellent mental and physical health. Urinalysis showed modest pyuria but negative urine cultures. The creatinine level in the blood was 1.1 mg/dL. A computed tomography scan revealed a calculus that was 11 × 12 × 67 mm^3^ in size with a density of 680 Hounsfield units at the right proximal ureter (Fig. 1A). Excretion was mildly decreased in the right kidney, but normal excretion was observed on the left side. After discussing the various treatment options with the patient, we concluded together that mini-ECIRS would be preferred.

Intravenous prophylactic antibiotics (cefuroxime) were administered during the induction period of general anesthesia. The patient was placed in the Galdakao-modified supine Valdivia position, in which the right flank was raised, and the buttock and leg were set in an asymmetrical lithotomy position. Two operating instruments were used simultaneously by two urologists: one used a miniature nephroscope, while the other used a flexible ureteroscope (fURS).

**Fig. 2:**
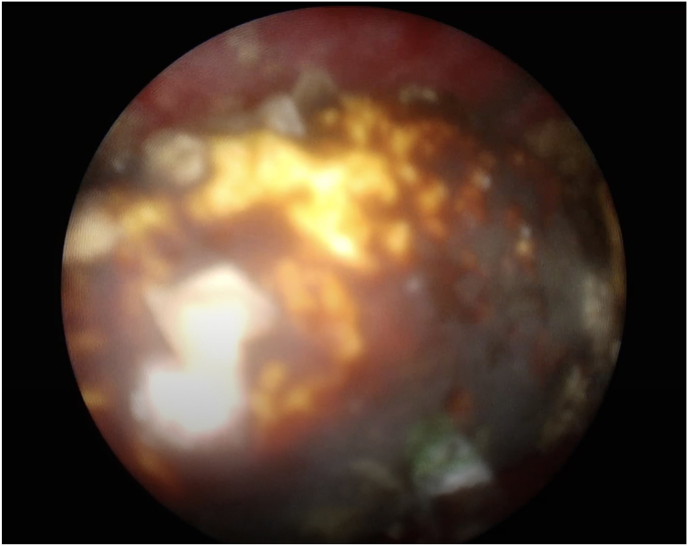
Miniature nephoscope view of the giant calculus.

**Fig. 3:**
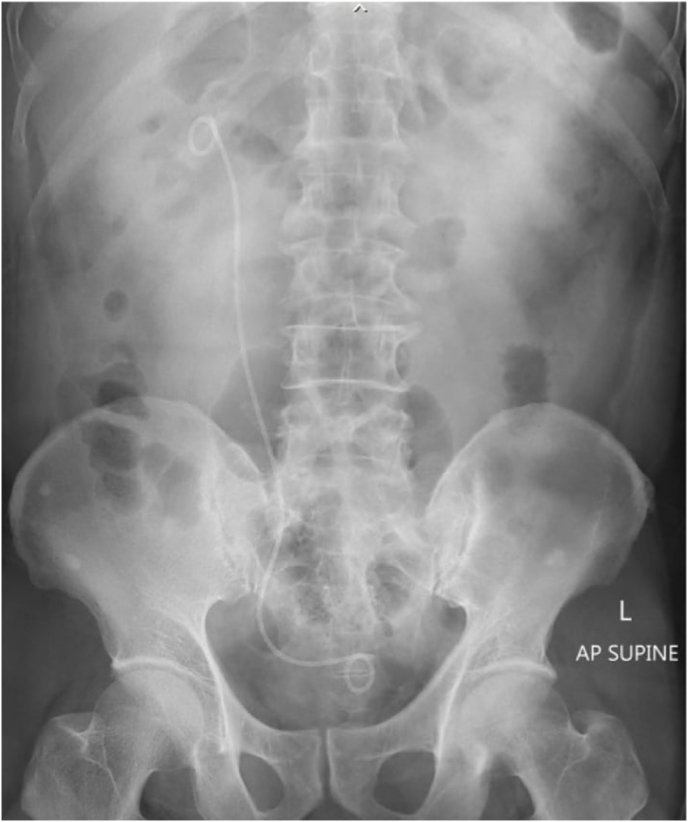
Post-operative abdominal plain radiography showing no evidence of residual calculi.

First, a cystoscopy was carried out to gain retrograde access, and a retrograde pyelogram was performed to delineate the ureter and pelvicalyceal system. Two hybrid guidewires (Sensor™, Boston Scientific, Natick, MA) were then inserted into the ureteric orifice to reach the renal pelvis. A digital fURS, LithoVue™ (Boston Scientific, Marlborough, MA), was introduced over one guidewire until it made contact with the giant calculus. The stone pushed retrogradely into the renal pelvis by advancing the fURS without difficulty (Fig. 1B). The second urologist performed an ultrasound-guided percutaneous puncture using a 18 G metallic needle point to the lower pole of the kidney. Tract dilatation was achieved over the guidewire to establish a 15 F working channel under direct visualization via the fURS. Laser lithotripsy was performed using the fragmentation technique with a 120 W holmium laser (Lumenis, San Jose, CA) and a 550 μm core laser fiber with energy of 1.5 J and a rate of 30 Hz (Fig. 2). Finally, all the pieces of stone were extracted with graspers through a 12 Fr nephroscope MIP-M system (Karl Storz, City, Germany) or by the Venturi effect. At the end of the procedure, a retrograde 6Fr double-J stent was placed. We obtained plain films of the abdomen to assess residual stones and confirm that the patient kept double-J stent in the appropriate position (Fig. 3). The patient made an uneventful postoperative recovery. The stent was removed cystoscopically at 6 weeks after discharge.

## 3 Discussion

Ureteral calculi are stones made up of crystals in the renal collecting system that then migrate down the ureter. They are more likely to become stuck in locations where the ureter is at its narrowest. In general, stones that are less than 4 mm in size are small enough to pass spontaneously in most patients. However, for stones greater than 7 mm in width, the probability of spontaneous passage is significantly lower.^2^ The best solution for a giant ureteric calculus is controversial. The open approach is the preferred choice for most surgeons; however, extracorporeal shock wave lithotripsy, transurethral lithotripsy, percutaneous nephrolithotomy (PCNL), and laparoscopic surgery have also been reported. Notwithstanding, there are no reports of mini-ECIRS for the management of this interesting calculus.

ECIRS was first described by Scoffone in 2008 to standardize the synergist procedure between retrograde intrarenal surgery and PCNL for large and/or complex urolithiasis with the aim of improving efficiency and safety.^3^ This combination technique facilitates lithotripsy while keeping the patient in a single position and accessing the collecting system only once. The distinguishing benefits of performing the double scopes demonstrated in our case were the relocation of the giant stone upward into the renal pelvis, ureteroscopy-assisted puncture, and the retrieval of any fragments of broken stone that scattered in the different calyx that evade multiple tracts and may cause related bleeding. Moreover, we employed the small access sheath (15 Fr) of a miniature nephroscope, which has significant advantages in terms of reducing the likelihood of kidney injury, bleeding, and the requirement for transfusion.^4^ The nephrostomy-free or “tubeless” option was considered appropriate in this case as it helps reduce postoperative pain, analgesia requirements, and hospital stay. We confirm the excellent results of the mini-ECIRS procedure by the absence of any pieces of residual stone in a plain films of the abdomen administered at post-surgery.

## 4 Conclusion

We successfully treated a giant ureteric calculus using mini-ECIRS. This novel technique is safe and feasible, with low morbidity.

## Declaration of competing interest

None.

## References

[1] Gupta N., Bansal U., Mahajan N. (2015). Giant ureteric calculus leading to autonephrectomy. Hellenic J Surg.

[2] Coll D.M., Varanelli M.J., Smith R.C. (2002). Relationship of spontaneous passage of ureteral calculi to stone size and location as revealed by unenhanced helical CT. Am J Roentgenol.

[3] Scoffone C.M., Cracco C.M., Cossu M., Grande S., Poggio M., Scarpa R.M. (2008). Endoscopic combined intrarenal surgery in Galdakao-modified supine Valdivia position: a new standard for percutaneous nephrolithotomy?. Eur Urol.

[4] Ketsuwan C., Phengsalae Y., Viseshsindh W., Ratanapornsompong W., Kiatprungvech N., Kongchareonsombat W. (2021). Implementation of supine percutaneous nephroscopic surgery to remove an upward migration of ureteral catheter in infancy: a case report. Res Rep Urol.

